# KLIFS: a structural kinase-ligand interaction database

**DOI:** 10.1093/nar/gkv1082

**Published:** 2015-10-22

**Authors:** Albert J. Kooistra, Georgi K. Kanev, Oscar P.J. van Linden, Rob Leurs, Iwan J.P. de Esch, Chris de Graaf

**Affiliations:** Division of Medicinal Chemistry, Amsterdam Institute for Molecules, Medicines and Systems (AIMMS), Vrije Universiteit Amsterdam, Amsterdam, 1081 HV, The Netherlands

## Abstract

Protein kinases play a crucial role in cell signaling and are important drug targets in several therapeutic areas. The KLIFS database contains detailed structural kinase-ligand interaction information derived from all (>2900) structures of catalytic domains of human and mouse protein kinases deposited in the Protein Data Bank in order to provide insights into the structural determinants of kinase-ligand binding and selectivity. The kinase structures have been processed in a consistent manner by systematically analyzing the structural features and molecular interaction fingerprints (IFPs) of a predefined set of 85 binding site residues with bound ligands. KLIFS has been completely rebuilt and extended (>65% more structures) since its first release as a data set, including: novel automated annotation methods for (i) the assessment of ligand-targeted subpockets and the analysis of (ii) DFG and (iii) αC-helix conformations; improved and automated protocols for (iv) the generation of sequence/structure alignments, (v) the curation of ligand atom and bond typing for accurate IFP analysis and (vi) weekly database updates. KLIFS is now accessible via a website (http://klifs.vu-compmedchem.nl) that provides a comprehensive visual presentation of different types of chemical, biological and structural chemogenomics data, and allows the user to easily access, compare, search and download the data.

## INTRODUCTION

**Figure 1.**
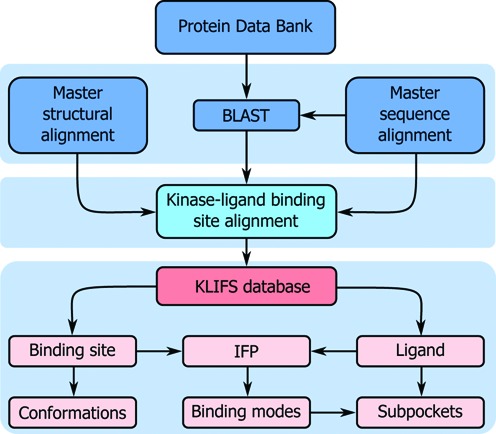
The data collection, processing, annotation and analysis workflow of KLIFS.

Protein kinases are enzymes that modulate the biological activity and expression of proteins by catalyzing the phosphorylation of serine, threonine or tyrosine residues. The 518 human protein kinases constitute one of the largest protein families encoded within the human genome and play essential roles in the majority of cell signal transduction pathways (1). Kinases have therefore become important drug targets for pharmaceutical intervention in several therapeutic areas, including oncology, immunology, neurology, cardiology and infectious diseases (2,3). The catalytic domains of kinases share a conserved structure, which poses a challenge for the development of small molecule drugs that can selectively target a well-defined set of kinases in order to obtain the desired (poly)pharmacological effects (4). Currently (August 12, 2015), 2899 structures of human and mouse catalytic kinase domains have been experimentally determined (2892 X-ray, 5 NMR and 2 EM structures; see Supplementary Table S1) that can offer insights into the structural determinants of kinase-ligand interaction and selectivity. We have collected, processed, annotated and analyzed all available structural kinase-ligand interaction information in a single, enriched and searchable web resource, KLIFS—Kinase-Ligand Interaction Fingerprints and Structures—database, to enable systematic comparison and analysis of the chemical and structural features of all available experimentally-determined protein kinase structures and their small molecule ligands (Figure 1).

**Figure 2.**
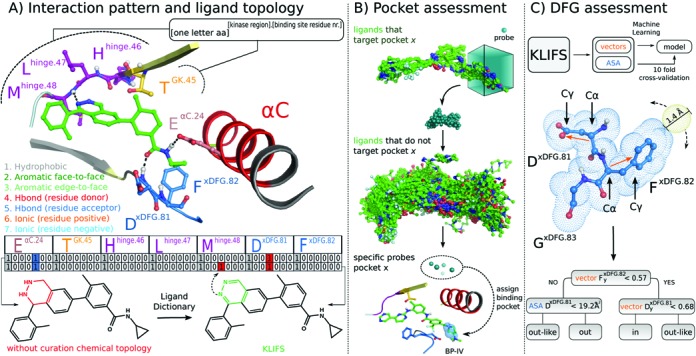
Annotation structural kinase-ligand interaction data in KLIFS. (**A**) Consistent structure-based kinase binding site residue annotation (top) and careful curation of the chemical topology and protonation of kinase ligands (bottom) enable the systematic analysis of kinase-ligand IFPs of kinase-ligand complexes (middle), illustrated for 7 of the 85 binding site residues of a phthalazine inhibitor (PDB ligand identifier: A17) bound p38a structure (PDB: 3DS6). (**B**) Automated assessment of ligand-targeted subpockets in protein kinase binding sites. Spatial probes (0.5 Å grid spacing) are: (i) placed around the conformations of kinase-bound ligands that are superposed according to the structural alignment of the corresponding binding sites; (ii) scored according to the ratio of close contacts (<1.0 Å) with training sets of ligands that bind a specific subpocket and ligands that do *not* bind this subpocket; (iii) highest ranked probes are selected as descriptors to assign subpockets for new kinase-ligand complexes. (**C**) A 10-fold cross-validated decision tree model derived from systematic analysis of the Accessible Surface Area (ASA) (32) of kinase binding site residues and vectors defining the directionality of D^xDFG.81^ and F^xDFG.82^ side chains enable the automatic assessment of the DFG conformation of kinase structures with an accuracy > 99%. The decision tree algorithm identified the ASA values of D^xDFG.81^ and y-values of D^xDFG.81^ and F^xDFG.82^ vectors (obtained by subtracting the coordinates of Cγ by Cα) as the most predictive descriptor combination for the discrimination of DFG conformations.

The initial version of KLIFS (5) was created based on a systematic analysis of all human kinase domain structures that were available in the Protein Data Bank (PDB) (6,7) at that point in time (1734 in total—August 9, 2012). The foundation of KLIFS was the definition of a consistent binding site encompassing 85 pocket residues that interacted with any bound kinase inhibitor within the catalytic front cleft, gate area and/or back cleft (type I, I$\frac{1}{2}$, II and III (8)) to allow for the systematic analysis of kinase-ligand interaction fingerprints (IFPs) (9) with different residues in the kinase binding site (Figure 2A) (5) in order to identify kinase (family) specific interaction features and classify ligands according to their binding modes. Accompanying this binding site definition was the introduction of a numbering scheme, in which each binding site residue is labeled according to the following scheme: [one letter amino acid]^[kinase region].[binding site residue number]^, e.g. the aspartic acid of the xDFG motif is labeled D^xDFG.81^ (Figure 2A). This numbering scheme is also used throughout this article.

Currently the amount of available human kinase structures has grown with 982 new structures to a total of 2738 in 3 years (August 12, 2015), covering 234 different kinases from all eight main kinase groups (AGC, CAMK, CK1, CMGC, STE, TK, TKL and other (1)), and including 1740 unique kinase inhibitors. This rapid increase emphasizes the need for KLIFS as a protein-family specific database that integrates different types of structural and chemical data. Since the first release of KLIFS we have continued our work with the aim to make our database up-to-date, and easily accessible for scientists with different backgrounds. This has resulted in a significantly extended database with new types of structural kinase-ligand interaction data, which is now publicly accessible and actively searchable via a user-friendly web interface (http://klifs.vu-compmedchem.nl). Moreover, automated weekly updates keep the database in sync with the Protein Data Bank. This new web-based version of KLIFS provides a clear and comprehensive visual presentation of different types of chemical, biological and structural chemogenomics data, and allows the user to easily access, compare and search the data to improve our understanding of the structural requirements of kinase-ligand interaction.

## CREATION OF KLIFS

### Data collection and preparation

All sequences from human (490) and mouse (525) eukaryotic protein kinases, the two most prevalent species in published kinase structures, are used as input for a BLAST query (10) against all structures released by the PDB (Figure 1). BLAST hits (≥70% sequence identity and an *e*-value ≤ 0.001) are selected for preparation and further processing. First the structures are prepared by splitting each structure into separate (alternate) models, if present, and individual chains after which each monomer is converted to atom and bond type annotated Tripos MOL2 format (to facilitate IFP analysis). Then the data in the Chemical Component Dictionary (11) are used to identify and correct errors in atom and bond types (Figure 2A) to allow for proper protonation of the molecules and accurate analysis of protein-ligand interactions, including H-bond interaction networks (12). Manual curation of the ligand topologies resulted in additional corrections (examples shown in Supplementary Figure S1).

### Data processing

A master sequence alignment (MSeq) of all human and mouse catalytic domains of eukaryotic protein kinases and a master structural alignment (MStruc) comprising three structures from each kinase group (Supplementary Table S2) were created with a focus on the binding pocket (5). The prepared monomers based on the BLAST search are subsequently processed using MOE (Chemical Computing Group Inc.) by performing a sequence alignment to the MSeq (Figure 1). The resulting sequence alignment is used to superpose each structure to the MStruc (Figure 1) based on residues of the catalytic loop, the DFG motif and the hinge region. This process gives a robust structural alignment of kinase binding sites as indicated by the low RMSD of 0.8 ± 0.1 Å for the superposing residues and an RSMD of 2.2 ± 0.2 Å for the full binding pocket (as defined by the 85 pocket residues) on average over all 2899 currently processed structures. Afterward, all individual elements (final sequence alignment, full complex, protein, pocket, orthosteric ligands (here defined as all ligands binding to the catalytic front cleft, gate area and/or back cleft), allosteric ligands (here defined as all ligands not fitting the orthosteric classification), waters, ions, cofactors, organometallics and alternative amino acids) are extracted and stored in MOL2 format.

### Data annotation and analysis

The extracted structure elements are used to analyze and annotate each structure (Figure 1). One of the key elements of KLIFS is the annotation of kinase-ligand interactions, which is performed by calculating the IFPs using the FingerPrintLib developed by Marcou and Rognan (9). An IFP (Figure 2A) encodes seven different interactions types (hydrophobic contact, aromatic face-to-face, aromatic edge-to-face, H-bond donor-acceptor, H-bond acceptor-donor, ionic positive-negative, ionic negative-positive) between each of the pocket residues and the ligand in a bit string as either present (1) or absent (0). If a structure has missing residues or gaps in the pocket, the IFP is corrected by inserting seven zeroes at the respective position to allow systematic comparison of all IFPs within KLIFS.

Based on the manual annotation of the binding modes of the ligands in the previous version of KLIFS with respect to specific (sub) pockets (5,13) we were able to train an automated method to identify which pockets are occupied by each bound ligand. This method annotates whether or not a ligand addresses any of the 3 major pockets (front cleft, gate area and back cleft) and the 12 subpockets (front pockets I and II, back pockets I-A, I-B, II-in, II-A-in, II-B-in, II-out, II-B, III, IV and V) by generating clusters of probes that accurately describe these pockets (Figure 2B). If a ligand contacts any of the probes for a specific (sub) pocket, the binding mode for that ligand is annotated as such (Figure 2B). Moreover, by analyzing all water molecules interacting with a ligand we were able to define 13 conserved water clusters (I1–I11 and 2 specific DFG-out clusters O1 and O2; Supplementary Figure S2). For each structure all waters within any of the clusters are extracted and their H-bond pattern with the ligand and/or protein is evaluated using IFP.

The conformation of the DFG-motif (side chain and backbone movement) determines if a kinase is in the active (DFG-in) or inactive (DFG-out) state or somewhere in between (DFG-outlike) (13,14). Based on the manually curated first version of KLIFS, decision tree machine learning (15) was applied and resulted in a rule-based method to automatically annotate the conformation (Figure 2C). The position of the αC-helix can also be classified into αC-in, αC-outlike or αC-out based on the distance between the Cα atoms of F^xDFG.82^ and E^αC.24^ (5,13). The conformation of the G-rich loop is annotated based on the angle of this loop compared to hinge region and catalytic loop, the distance between the catalytic loop and the G-rich loop, as well as the rotation of the G-rich loop compared to the catalytic loop (Supplementary Figure S3). Furthermore, all missing residue atoms in the pocket are logged and the pocket residues are checked for mutations by comparing the residues to the sequence in the Uniprot database (16). Finally, the quality of the structure with respect to the binding pocket as well as the quality of the processing by KLIFS is estimated with a so-called quality score that runs from 0 (bad) to 10 (flawless) and combines the resulting RMSDs relative to the MStruc with the number of missing residues and missing atoms (Supplementary Figure S4).

## THE KLIFS WEBSITE

In order to allow researchers to easily browse, search, visualize and download data from KLIFS we have built a user-friendly and feature-rich web interface with a clear organization that gives access to all underlying data.

### Organization

**Figure 3.**
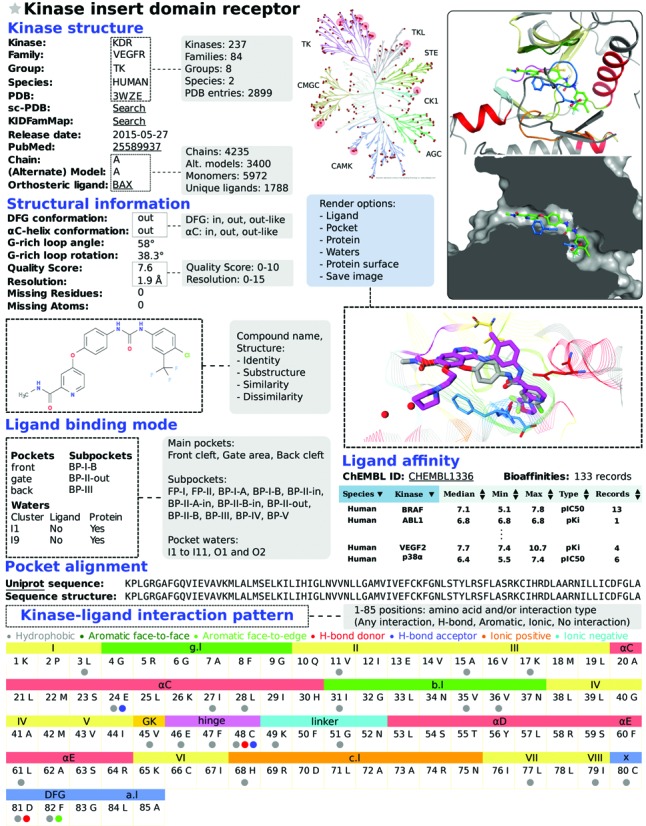
KLIFS entry example (sorafenib (BAX) bound KDR, PDB: 3WZE), schematic overview of KLIFS website search options (gray boxes) and 3D kinase-ligand binding mode visualization (overlay sorafenib gray carbon atoms, ligand from search example PDB: 2BAK magenta carbon atoms). All kinases with an entry in KLIFS are indicated in the interactive human kinome (red dots), and search results can also be highlighted. Top right panels: sorafenib binding mode views showing (color-coded) secondary structure elements, sliced binding site surface and user-customizable 3D-rendering settings (blue box). Further details regarding structural information, ligand search/binding mode/affinity, pocket alignment/kinase-ligand interaction pattern browse/search options are explained in the text.

The web interface contains six main pages: *Browse*, *Search*, *Data charts*, *Favorites*, *Tutorial* and *FAQ*. *Browse* allows users to go through a complete overview of all structures. *Search* gives access to multiple search options, which can be used to focus on the most relevant structures. A detailed overview page is provided for each structure and can be accessed when browsing or searching KLIFS (Figure 3). Via *Data charts* users can compare different structure attributes and visualize them in histograms. Via *Favorites* one can keep track of specific structures throughout the website and it also enables the comparison of ligand binding modes via the integrated WebGL 3D-viewer (iview (17)). Finally, *Tutorial* provides an overview of the website and several usage examples, while *FAQ* contains answers to (anticipated) frequently asked questions.

### Features of the website

#### Searching

There are several search options embedded within the KLIFS website that can be used simultaneously to create a highly versatile way of focusing the available data (Figure 3). On the home page the latest entries are listed and the interactive HTML5 human kinome (based on Kinome Render (18)) can be used to search for specific kinases. From any page the *PDB search* option is available that can be used to quickly find a specific structure or ligand using their PDB-codes (e.g. structure 2XYU or ligand Q9G). The options on the main search page have been divided into nine categories: (i) ligand structure, (ii) kinase classification, (iii) structural annotation, (iv) binding mode, (v) pocket waters, (vi) structure properties, (vii) ligand properties, (viii) binding pocket composition (sequence) and (ix) ligand-kinase interaction pattern (Figure 3). The *ligand structure* search option allows the users to provide the molecular SMILES, draw a chemical structure or retrieve a molecular structure based on the name (via the HTML5 Molecule editor, MolSoft LLC). This ligand structure can subsequently be used to search for structures containing this exact ligand (or tautomers thereof (19)), a (dis)similar ligand (based on Morgan (20) or MACCS (MDL Information Systems, Inc.) fingerprint similarity) or a substructure (using RDKit: Open-source chemoinformatics; http://www.rdkit.org). The kinase classification search option allows for the selection of specific kinase families, kinase groups or organisms. The structural annotation options allow filtering based on DFG and αC-helix conformation, and whether the structure is ligand-bound or not. Via the binding mode selection options the user can carefully define whether the ligand does or does not bind to a specific (combination of) subpocket(s) in the protein kinase binding site (Figures 2B and 3). Moreover, one can search for the presence or absence of water in one or more of the 13 water clusters and their H-bonding pattern. The structure properties give the user the possibility to select requirements for the G-rich loop conformation, quality score and the resolution of the kinase structure. The ligand properties search allows for the manual selection of physicochemical properties filter or the selection of one of the 4 presets: the rule-of-five, the rule-of-three, fragment-like and lead-like (21,22). The binding pocket composition feature provides the option to select specific amino acid at specific residue positions in the pocket (e.g. a threonine (T) on position 45, i.e. the gatekeeper, e.g. see Figure 2A). Finally, the interaction pattern search option gives access to the ligand-kinase interactions and enables the user to select which interactions the bound ligand should have with specific binding pocket residues. Additionally, all search results can be projected onto the interactive human kinome.

#### Viewing

Each structure in KLIFS has a detailed information page that presents all information within the KLIFS database regarding this specific structure (Figure 3). First all information regarding the kinase type (names as defined by the HUGO Gene Nomenclature Committee (23) and Uniprot (16)), the (crystal) structure, and (if present) the orthosteric and/or allosteric ligand is presented including a 2D chemical representation, images of the binding pocket (created with Pymol, Schrödinger LLC.) and a link to the integrated 3D-viewer (17). Second, all structural information is presented: DFG, αC-helix and G-rich loop conformation, quality score, resolution, missing residues and atoms. Third, if a ligand is present in the binding site all targeted (sub) pockets are listed as well as all pocket waters within any of the 13 water clusters. Fourth, kinase records in ChEMBL (*K*_i_ or IC_50_, confidence≥8) for the bound ligand are listed if present. Fifth, the sequence of binding site of the structure is compared to the sequence from Uniprot. And finally, the interaction profile of the ligand with the binding site is shown in which each interaction with each of the 85 binding site residues is highlighted. Moreover, the interaction profile of the selected structure can be used to find other kinase-ligand complexes with a similar interaction profile. To easily allow access to additional relevant information, links have been integrated to the following external databases: ChEMBL (24), PDB (6,7), sc-PDB (25), KIDFamMap (26), Uniprot (16) and PubMed (NCBI).

#### Downloading

The results of each search query can be exported to an Excel or a CSV file containing all details as listed in the search results overview. Moreover, via the ‘Download Structures’ option all annotated and structural data can be downloaded as a MOE database or as a compressed package comprising all processed MOL2 files and a CSV detailing all annotation data. This enables researchers to use their software of choice for the comparison and analysis of the structures.

### Examples of search queries

#### Design of selective BTK inhibitors

Shi et al. (27) synthesized a purine derivative (PDB: 4NWM) with (high) affinity for BTK, but also for JAK2, Flt2 and ITK. Subsequently, the authors designed new analogues with the aim to target the gatekeeper region in order to obtain BTK selectivity as BTK has a relatively small T gatekeeper compared to the M/F^GK.45^ of the off-target kinases. The most selective compound was obtained by attaching a 2-phenylethyl group to the 9 position of the purine. A search of the current KLIFS database for a T^GK.45^ and a ligand targeting both the front cleft and gate area, but *not* the back cleft yielded 98 structures that can be used to design ligands with similar (BTK selective) interaction features. Limiting the search to a purine scaffold resulted in only 2 entries (PDB: 2BDJ, 4GK2), of which 2BDJ contained a bound ligand with a 2-phenolethyl moiety on the 9 position of the purine, allowing for an additional H-bond with D^xDFG.81^ (see ligand overlay in Supplementary Figure S5).

#### Water-mediated H-bonding and pocket composition

Levinson and Boxer (28) identified a strong correlation between conserved water molecules, cavity-lining residues and the selectivity profile of bosutinib. The presence of primarily water W1 (cluster I5 in KLIFS), but also water W2 (cluster I4 in KLIFS) allowed for a water-mediated H-bond between bosutinib and the DFG-motif. A KLIFS search for both waters I4 and I5 yielded 648 structures (92 different kinases). A more detailed search for the crucial I5 water and the key binding site residues T^GK.45^ (bulky amino acids prevent water-mediated H-bonding), V^b.I.36^ (a leucine substitution reduces affinity) and A^xDFG.80^ (larger amino acids obstruct access to water I5) resulted in only 6 unique kinases (29 structures), which all have (a predominantly high) affinity for bosutinib.

#### Diverse molecules with similar interaction patterns

Finding dissimilar molecules with similar interaction patterns can be interesting from a drug design point of view (e.g. scaffold hopping) (29,30). Starting from a sorafenib-KDR complex (PDB: 3WZE, Figure 3) an interaction similarity search was performed (similarity ≥ 0.75) resulting in 51 entries (44 unique ligands in 17 different kinases). Further refinement by additionally performing a dissimilarity search to sorafenib (Morgan similarity ≤ 0.25) limited the result to 21 structures. One of the identified structures (PDB: 2BAK, IFP similarity = 0.77) was of p38a in complex with an anilinoquinazoline inhibitor (see integrated 3D viewer overlay in Figure 3), which has a comparable affinity for p38a as sorafenib (31).

## CONCLUSIONS AND OUTLOOK

With this new version KLIFS has grown from a downloadable data set to a fully featured database with a user-friendly, yet versatile, web interface. On the back end the sequence alignment, structure superposing, structure processing and kinase-ligand interaction analysis protocols have been improved and new methods have been developed for the automated annotation of subpocket-targeting by ligands, DFG and αC-helix conformations and the curation of ligand topologies for accurate kinase-ligand interaction analysis. Moreover, KLIFS will be updated weekly in sync with the Protein Data Bank to keep up with the continuous growth in published catalytic kinase domain structures (averaging 380 new structures per year over the past three years). The new front end, the web interface, provides easy access to all data and has additional features like (combined) search options based on kinase-ligand interactions, binding site sequence and ligand features, as well as (3D) viewing and rendering options to interactively analyze and compare the structures of kinase-ligand complexes.

We believe that this database will prove to be a valuable resource of structural, molecular, biological and chemogenomics kinase data. For example, computational chemists and bioinformaticians can use this database for advanced and extensive structural and sequence analyses, modeling and (structure-based) machine learning approaches, structural biologists can compare kinase structures and validate their own structures, pharmacologists can related their data (e.g. mutation data) to the available structures, and medicinal chemists can use this database to gain new structural insights and for the rational design of novel (selective) kinase inhibitors.
